# Gearing health systems for universal health coverage

**DOI:** 10.3389/frhs.2023.1200238

**Published:** 2023-09-21

**Authors:** Marlon E. Cerf

**Affiliations:** ^1^ Grants, Innovation and Product Development, South African Medical Research Council, Cape Town, South Africa; ^2^ Biomedical Research and Innovation Platform, South African Medical Research Council, Cape Town, South Africa

**Keywords:** global health strategy, healthcare, health systems strengthening, optimal health services, Sustainable Development Goals

## Abstract

Universal health coverage requires adequate and sustainable resourcing, which includes human capital, finance and infrastructure for its realization and sustainability. Well-functioning health systems enable health service delivery and therefore need to be either adequately or optimally geared—prepared and equipped—for service delivery to advance universal health coverage. Adequately geared health systems have sufficient capacity and capability per resourcing levels whereas optimally geared health systems achieve the best possible capacity and capability per resourcing levels. Adequately or optimally geared health systems help to mitigate health system constraints, challenges and inefficiencies. Effective, efficient, equitable, robust, resilient and responsive health systems are elements for implementing and realizing universal health coverage and are embedded and aligned to a global people-centric health strategy. These elements build, enhance and sustain health systems to advance universal health coverage. Effective and efficient health systems encompass continuous improvement and high performance for providing quality healthcare. Robust and resilient health systems provide a supportive and enabling environment for health service delivery. Responsive and equitable health systems prioritize people and access to healthcare. Efforts should be made to design, construct, re-define, refine and optimize health systems that are effective, efficient, equitable, robust, resilient and responsive to deliver decent quality healthcare for all.

## 1. Introduction

The Sustainable Development Goals (SDGs) focus on global societal, environmental, economic and health advancement, by addressing key challenges that constrain progress, for the benefit of global citizens. The SDGs are inclusive, integrated, interdependent and equitable goals that represent global action for sustainable development—these ambitious goals encompass people, planet, prosperity, peace and partnership (1, 2). SDG 3, good health for all, aims to advance global health by focusing on targets to reduce morbidity and mortality i.e., to improve the health and well-being of all. SDG 3 encompasses all the health goals (3.1–3.9 and 3.A-3.D) that collectively aspire to improve health outcomes by targeting major diseases, with SDG 3.8 aiming for universal health coverage (UHC). UHC is people-centric and strives for access to quality, affordable and equitable healthcare i.e., healthcare for all people without financial hardship (1). In addition, UHC is a major global political and ethical health goal embracing the key ethical concepts of fairness, equity, and benefit, concomitant with solidarity justifying the mutual determination for achieving UHC (3) nationally, regionally, continentally and globally (4). Universal health coverage and equity should be prioritized to combat avoidable and unfair morbidity and mortality (5) that afflict the most vulnerable and underserved people. UHC can be advanced through improved efficiency, equitable resource distribution, accountability and transparency (6). Further, high inequalities in health resources in favor of advantaged populations constrain equal access to healthcare and the realization of better health outcomes (7). The health system components of UHC are the health workforce, health facilities and health financing (health resourcing), health service delivery (health systems enable health service delivery), communications networks, health technologies and information systems (healthcare enablers for health system effectiveness and efficiencies), quality assurance mechanisms (for ensuring the delivery of quality healthcare i.e., health system effectiveness), and governance and legislation (for reinforcing robust and resilient health systems) (8) which represent resourcing. Sufficient health resourcing is required for equipping and preparing health systems, i.e., gearing health systems, for delivering quality and timely healthcare and is shaped by building blocks, i.e., elements, to build, enhance and sustain health systems, which will be presented in the article.

UHC is people-centric and the World Health Organization (WHO) global strategy on integrated people-centric health services lays out the fundamentals of progressive health services for modern needs (9). Key principles are to embed a person-centric vision, which relates to the patient experience and outcomes, healthcare access to all, given that ∼1 billion people cannot access decent healthcare; a focus on the life course (from fetal life to ageing); and effective, efficient, safe, and timely healthcare delivery (quality healthcare) (2, 9). The key deliverable from the WHO global strategy on integrated people-centric health services is the implementation of UHC (9). Therefore, health systems need to be adequately or optimally geared, dependent on the resourcing availability, to realize people-centric health service delivery.

Good health and universal coverage for citizens captures the essence of SDG 3, based on the people-centric global health services strategy (9), which is enabled by adequately or optimally geared health systems to provide decent, affordable and equitable healthcare. Good health services are comprehensive, appropriate for the target population's needs, and cover preventative, curative, palliative and rehabilitative healthcare, and health promotion (10). To advance and enable UHC, effective, efficient (4), equitable, responsive, robust and resilient (9) health systems are requisite, but delivery, access and quality of healthcare is variable, within and between countries. Access to decent health services is a basic human right and therefore barriers to care need to be mitigated.

The article presents elements for building, enhancing and sustaining health systems that serve to advance UHC. These elements enable gearing, which is to equip and prepare health systems, to meet demands and are aligned to UHC pillars: provide quality (decent) and accessible (equitable) healthcare to people without the risk of financial hardship (affordable); and support a people-centric global health strategy. Some health systems challenges are discussed with insights presented on overcoming some health systems inefficiencies, followed by an overview of the WHO's global health strategy, then the gearing and elements of health systems are unpacked and related to realizing UHC for better health outcomes.

## 2. Health systems

### 2.1. Health system constraints and challenges

Health systems should achieve good health, responsiveness and financial protection (6, 11). However, there are major barriers to access to healthcare, and sound plans and policies with clear road maps are imperative for strengthening health systems in low- and middle-income countries (LMICs), especially in Africa, and to address the socioeconomic determinants of health (12). Building, enhancing and sustaining robust health systems are necessary to achieve health targets and better health outcomes. Health systems are either inadequately, adequately or optimally geared to deliver health services (Supplementary Material Table S1), dependent on health resourcing which varies within and between countries. Inadequately resourced health systems are unable to deliver basic health services (Supplementary Material Table S1). To achieve adequately or optimally geared health systems for implementing a people-centric global health strategy, barriers need to be overcome with the best use of the available resources. Governance and accountability often require strengthening in ineffective and inefficient health systems. Although absolute zero wastage is somewhat unrealistic, there are levers to improve system efficiencies e.g., adopting a preference for generic drugs and combatting corruption, thereby releasing resources and reducing the forecasted costs (13). Health systems efficiencies are particularly critical in LMICs that are under-resourced and rely on cost-effectiveness, while operating in these poorly resourced settings, to maintain and enhance service delivery i.e., to be effective. Delivering healthcare in poorly resourced settings places some to severe strain on operational efficiencies. Therefore, sound financial management and effective and efficient implementation capacity are necessary to utilize resources optimally (13) for advancing UHC. For accountability and efficiency, health services need to be responsibly and well-managed to optimize the utilization of resources for the delivery of quality healthcare. Corruption and wasteful or sub-optimal use of often limited resources diminishes cost-efficiencies and the effective delivery of healthcare. All people have a vested interest in health. Thus, the more health services achieved per currency unit, e.g., dollar, spent, the better for all people. Adequately or optimally geared health systems (Supplementary Material Table S1) help to extend the health services delivered for every currency unit spent.

About 20%–40% of health resources are wasted, thus reducing this waste improves the ability of health systems to provide quality services and improve health outcomes, i.e., improved efficiency (14). Health sector corruption derails the advances in population health, social justice, shared prosperity and sustainable development, all core pillars of the SDGs (15) and weakens health systems. Corruption due to ineffective governance presents a major cause of health system inefficiencies (16), and ∼10%–25% of public health spending vanishes due to corruption. Corruption impacts the costs of health service delivery as it reduces the volume yet augments the cost of health services which subsequently translates into adverse health (17) and economic outcomes as money is lost to corruption instead of being allocated to health resources, systems and services. Therefore, transparent funding and spending on health is required and any deviations require prompt and proper motivation or remedial action. This ultimately benefits health service delivery for better patient outcomes. Efforts to prevent corruption need to begin with international consensus and recognizing the unique and destructive consequences of health sector corruption (15). In addition, health sector corruption often extends into other sectors, given the integrated nature and network of suppliers and other actors in public and private sectors. Reducing corruption frees up resources to enable better healthcare access and delivery. Mitigating corruption particularly benefits health system effectiveness and efficiencies.

### 2.2. Overcoming health system inefficiencies

Improved operational processes can help to reduce slack in health systems, in alignment with health system effectiveness and efficiency. This requires the input of reliable information through analyses of reliable, real-time data, thereby enhancing health systems' efficiencies, improving health (i.e., managerial and clinical) governance and strengthening health systems. Weak health resourcing capacity in LMICs increase the costs of implementing improvements, i.e., building, enhancing and sustaining health systems, and the prevailing inefficiencies often persist in future systems (13). Designing, constructing, re-configuring and re-aligning health systems for adequate or optimal gearing allows (i) a focus on primary and community healthcare with continuous improvement informed by timely, reliable data that confer some financial protection (quality and people-centric healthcare) and (ii) better affordability for the most vulnerable and underserved people, thereby enhancing quality and equity (18) (affordable and equitable healthcare) to advance UHC.

Sustainable funding is key for realizing UHC. Health financing strategies are generating more health funds, establishing larger pools to fund health, and realizing more health outputs for the funds invested (14). Countries can raise health funds by greater efficiency in revenue collection, reprioritizing government budgets (e.g., spend 15% of the budget on health) and introducing innovative financing (such as taxes on unhealthy foods) (14). Critical areas of health financing are raising sufficient health capital, removing financial barriers to access and reducing financial risks of illness, and making better use of available resources (14), which all contribute to advancing UHC.

Despite health system inefficiencies, adequate health resourcing in response to patients' needs will help to improve their experiences and outcomes. Striving towards adequately or optimally geared health systems strengthens and enables health systems towards advancing UHC. To achieve adequately to optimally geared health systems, specific elements—viz. effective, efficient, equitable, robust, resilient and responsive health systems—will be discussed. However, the global directions for health service delivery will first be presented to provide more context.

## 3. Global directions for health service delivery, health system elements, and adequately or optimally geared health systems

Understanding global health strategy is important for contextualizing health service delivery, through adequately or optimally geared health systems, in the context of SDG 3 for good health for all, and for UHC. UHC encompasses access to quality, affordable and equitable health services and can be leveraged to improve health and financial well-being and equity (19). UHC relies on adequately or optimally geared health systems for service delivery to provide all people with decent healthcare.

The WHO's people-centric and integrated global health strategy focuses on people-centric and integrated health service delivery, which reflects UHC, and has five interdependent strategic directions (9). First is providing better services, especially vulnerable and underserved people, to enable them to make better health decisions by empowering and engaging them (9). Better services resonate with the continuous improvement to achieve greater operational and cost efficiencies (health systems efficiencies) and higher functionality and performance (health systems effectiveness) for better service delivery. This reflects quality healthcare. Vulnerable and underserved people viz. poor, elderly and disabled people, mothers and children, including rural residents with limited access to health and basic services, should benefit most from UHC. This is aligned to equitable healthcare that opens access to vulnerable and underserved people. Health promotion is a tool to reach vulnerable and underserved communities and guide better health decisions for improved health outcomes. Engagement at the community level should be most effective in LMICs, where people can inform health workers of key health issues in the community. This will provide insights into existing and emerging morbidities and inform health system responsiveness. Every person has a right to decent healthcare. The first direction aligns with health system effectiveness, efficiency, equity and responsiveness.

The second direction focuses on strengthening governance and accountability and is achieved by adopting a robust, coherent, integrated approach to realize the harmonization and alignment of programs (9). For health systems, good governance mitigates against corruption, improves operational efficiencies and enables cost-effective health service delivery. Good health, i.e., managerial and clinical governance, fosters health systems strengthening. Aspects of good health governance encompass oversight, policy guidance, collaboration, regulation, accountability and system design and require a human rights approach, technical and political action, and attention to corruption (20). Clinical governance is defined by healthcare providers' accountability for continuously improving and safeguarding the quality of their services to achieve and maintain clinical service excellence (21–23). Clinical practice guidelines are typically evidence-based and informed by rigorous methodology (24), and can enhance clinical effectiveness and serve to assess quality assurance, as they are primarily designed to improve processes and outcomes of health interventions (25). The second direction aligns with health system effectiveness and efficiency. Technology is an enabler of adequately or optimally geared health systems. The application of enabling technologies is critical for facilitating governance and accountability. Integrated data management systems also help to obtain reliable, real-time information to plan and organize health resources e.g., number and skill levels of the health workers required, greatest disease burdens and stockouts of medication. This can identify constraints in health programs and harness and refine them for greater impact. Good governance is supported by robust health systems that enable resilience i.e., can absorb fluctuating health demands. With robust and durable health systems, health services are delivered effectively and efficiently which supports good health governance.

Thirdly, reorienting the model of care by prioritizing primary and community healthcare and the co-production of health is the direction; this will build stronger primary health systems and shift the focus towards outpatient care (9) which aligns with health system responsiveness. Responsive health systems focus on in- and out-patients’ health demands. For in-patients, responsive health systems evolve and are strengthened and adaptable for better treatment for patients. For out-patients, primary healthcare that focuses at the individual and community levels, through health promotion and disease prevention, relieves health systems. This helps to enhance health service delivery (health system effectiveness) as focus can shift to address health systems' challenges and improve health system efficiencies.

The fourth direction refers to coordinating services with a focus on delivery of healthcare through the alignment and harmonization of processes at every level of care and across sectors (9) (e.g., social services and education) to align with efficiencies for greater effectiveness. Highly coordinated and harmonized health services allow for early detection and rapid response to health crises i.e., enable better responsiveness. Africa has >100 outbreaks and other health emergencies annually, that translates into high morbidity, mortality, disability and socioeconomic disruptions that weaken already fragile health systems (18). Thus, having responsive health systems are critical for countering outbreaks, i.e., pandemic preparedness, while still providing necessary and decent healthcare. Pandemic preparedness requires continuous planning, exercising, revising and translating into national and subnational pandemic preparedness action and response plans, that entail regular reviewing and revision, based on information and experiences from previous outbreaks and pandemics, or from simulations (26). The WHO's Preparedness and Resilience for Emerging Threats Initiative (PRET) focuses on transmission and not specific diseases and has a monitoring framework for updating preparedness plans for prioritizing actions; increasing pandemic preparedness planning through greater stakeholder connectivity, coordination and cooperation; and dedicating sustainable financing, investments and monitoring of pandemic preparedness (27). Health systems need to adopt such frameworks to improve responsiveness. Responsive health systems also require coordinated public-private-philanthropy partnerships to support and improve quality and access to care.

The global Covid-19 pandemic infected and affected millions to billions of people globally and eroded health gains and economic sustainability. These adverse health and economic effects will take years to decades for recovery from the individual, national and global levels, particularly in LMICs. Further, many patients were unable to access regular healthcare as addressing outbreaks took priority which rendered them susceptible to deteriorating health and potential co- and multi-morbidities with infectious diseases. Therefore, developing capacities and capabilities for early detection, rapid response and prompt recovery is essential, as is strengthening health systems (18) given the global threat, unpredictability and rapid transmission of outbreaks. Health systems should therefore be equipped to respond to outbreaks and maintain reasonable service levels. It is critical for countries to prepare as best possible for future outbreaks given the high morbidity and mortality, globalized nature of transmission, and health resourcing and economic burden. The poorest resourced countries are particularly susceptible to health and economic collapse when outbreaks occur, with outbreak cases managed through already fragile health systems against a background of multiple and diverse diseases, multi-morbidities, polypharmacy and low health human capital, budgets, medication and infrastructure.

The final direction is creating an enabling environment to effect strategic directions 1–4 (9). This requires the consolidation of stakeholders for transformational change; legislative frameworks, financial arrangements, workforce reorientation and public policy making, all in support for realizing people-centric and integrated health service delivery through UHC. Robust and resilient health systems provide a supportive and enabling environment to realize UHC. The five strategic directions are interdependent and require simultaneous adoption to achieve success (9). Further, the five strategic directions collectively construct more effective health systems; therefore, slow progress in one direction may undermine progress made in the other directions (9). For creating an enabling environment in support of a people-centric global health strategy, it is imperative to realize effective, efficient, equitable, robust, resilient and responsive health systems that are adequately or optimally geared. These health systems elements are embedded and straddle these five global health directions, to enable better health service delivery and access for the benefit of people on the path towards UHC.

## 4. Gearing health systems for universal health coverage

Health systems support health service delivery—the better the health system, the better the healthcare provided. UHC requires adequate and sustainable resourcing, which includes human capital, finance and infrastructure, for its realization and sustainability. UHC also requires health systems strengthening. Health systems therefore need to be geared, i.e., equipped and prepared, through sufficient and sustainable resourcing to enable and augment health service delivery. Sufficient health resourcing refers to sufficient numbers of suitably skilled health workers, financial resources, medication and supplies, and well-maintained health facilities, equipment and infrastructure to sufficiently gear for health service delivery (Supplementary Material Table S1).

UHC aims to provide a minimum package of health services (8). The health resourcing levels determine the standard of care i.e., the minimum package of health services. With inadequate resourcing (Supplementary Material Table S1), health systems cannot deliver decent healthcare rendering the health systems ineffective. To achieve UHC, adequately or optimally geared health systems (Supplementary Material Table S1) are required to deliver the standard level of care. The focus is therefore on adequately geared health systems that enable the delivery of basic healthcare—delivering the basic standard level of care or minimum package of health services; and on optimally geared health systems that enable the delivery of the decent healthcare, beyond the basic standard level of care (Supplementary Material Table S1). Sufficient to ample health resourcing are the levers for achieving adequate and optimal health systems, respectively (Supplementary Material Table S1). Adequately geared health systems refer to sufficient health system capacity and capability, determined by the sufficient health resourcing to deliver health services i.e., people-centric quality, affordable and equitable healthcare. Adequately geared health systems can meet basic UHC, i.e., the minimum package of care, and operate at the threshold of minimal health resourcing for achieving the minimum package of care. Optimally geared health systems are defined as the best possible health system capacity and capability, given the health resourcing, to deliver health services i.e., people-centric quality, affordable and equitable healthcare. Optimally geared health systems meet and exceed UHC's minimum package of health services as the health resources are optimally utilized i.e., health resources are utilized as effectively and efficiently as possible to deliver the best possible quality health services.

## 5. Health system elements for gearing health systems to enable universal health coverage

To achieve SDG 3′s targets, including UHC, it is important to design, establish and maintain effective, efficient, equitable, robust, resilient and responsive (E3R3) health systems that are adequately or optimally geared and resourced. These elements gear and help to build, enhance and sustain health systems and represent key foundational and purposeful elements i.e., fundamental health system elements, or building blocks, that prepare and equip health systems for UHC.

**Figure 1:**
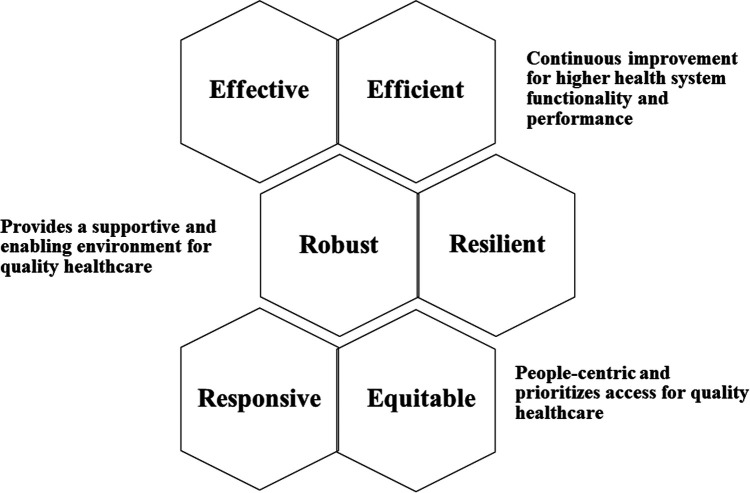
Health systems elements interlinkages.

The health system elements are depicted in Figure 1, are interlinked, and require further definition. The purpose and contribution of these elements for health service delivery and achieving UHC are described in Table 1. Quality healthcare refers to effective, safe, people-centric and timely delivery of health services (10). Effective and efficient health systems translate into better quality healthcare. Effectiveness refers to organizing plans and setting targets to achieve goals (28)—health system effectiveness (E1) requires the embedding of the elements, resources to deliver routine, quality, affordable, accessible and timely healthcare. Improving health systems' performance are determined by effectiveness in delivery, efficacy, costs, cost effectiveness, intervention response and quality of care (25). Health system effectiveness strives for higher functionality and performance for improved service delivery, which is dependent on the available resources. Efficiency refers to achieving high performance given the resources (29), e.g., personnel, time, funds and infrastructure; for health systems, efficiency is achieved by realizing UHC at optimized resourcing levels. Efficiency is a measure of the quality and/or quantity of output (i.e., health outcomes or services) per given level of input (i.e., cost), thus efficiency gains could help to contain costs by reducing the costs of health service delivery and can be achieved by extending health coverage at the same cost (14). Health system efficiencies (E2) focus on continuous improvement that drives and achieves greater operational and cost efficiencies. This aligns with providing decent or quality healthcare. Efficiency is achieved by optimizing the outputs per input unit invested (29). Countries can improve health systems efficiencies by releasing resources for coverage for more people and providing more services but this is challenging with increasing health costs and higher demands (14). Health system effectiveness and efficiency enable continuous improvement and higher performance (Table 1). Equitable healthcare (E3) is embedded in UHC that aims for access to all, so that vulnerable and underserved people have free access to decent healthcare. Health systems should be designed to cover patients and healthy people across all social, economic and geographic groups (10) i.e., be accessible to all people as UHC envisions.

**Table 1:** Health system elements for enabling universal health coverage.

Health system element	Purpose	Enabling universal health coverage
Effective (E1)	Higher health systems functionality and performance to provide quality health services	Improved service delivery to meet health demands
Efficient (E2)	Continuously improve operational, resourcing and cost efficiencies to provide quality health services	Better to optimal use of resources to provide quality healthcare
Equitable (E3)	Reach vulnerable and underserved people to enable access to healthcare	Better and greater access for all people to healthcare
Robust (R1)	Provide necessary resources to enable health service delivery	Mitigate interruptions in health services to ensure that reasonable delivery and access are maintained
Resilient (R2)	Meet dynamic and challenging health demands to maintain quality and access to health services	Agility, adaption and evolution to maintain steady health service delivery i.e., continuity
Responsive (R3)	Address people, community, district, sub-national and national health demands, i.e., people-centric health services to deliver relevant quality healthcare	Optimal resourcing informed by population needs for quality healthcare and coverage

Strengthening health systems aligns with robustness. Health system strengthening refers to meaningful and purposeful actions to enhance performance (30), i.e., effectiveness, and encompasses equity, quality, efficiency, resilience, accountability and sustainability, aligned with most national health plans, strategies and policies (31). Robust (R1) health systems require suitably trained and motivated health workers, adequate and well-maintained infrastructure, sufficient and reliable medical and technology supplies, sufficient funding, good health plans and evidence-based policies (32). Functioning health systems are therefore configured on people, institutions and resources to improve, maintain or restore population health (31). For health systems to be effective and efficient, they need to be robust and durable to enable resilience (R2), i.e., absorb fluctuating health demands and shocks to health systems. Health service demands can increase seasonally (e.g., colds and flu in winter) and during outbreaks (e.g., viruses such as Ebola and coronavirus) that shock health systems. Resilient health systems can absorb these shocks while still delivering decent healthcare i.e., maintaining healthcare continuity. Health system resilience is the ability to absorb disruptions, to adapt and respond with the requisite services (33) while maintaining core functions and serving the ongoing and acute care needs of communities and is supported by robust health systems. Further, health system resilience enables responsiveness. In addition, health system resilience aims to generate positive physical and mental health and well-being outcomes for all people, including vulnerable and underserved people (34). Thus health system resilience is closely associated with health system responsiveness, particularly in phases of high demand, as resilience is often requisite to effectively deliver health services. Efficiencies are derived from robust and effective health systems—with good health governance, efficiencies in health service delivery are realized. Robust health systems are closely linked to resilient health systems (R2) that are agile and adaptable to optimally reconfigure to meet dynamic and challenging health demands. Responding to healthcare demands reflects responsive health systems (R3), that are people-centric and focus on addressing people, community, district, sub-national and national health demands. Health system responsiveness aims for fair and equitable provision of health services, irrespective of social status (35), which should target the most vulnerable and underserved people. Further, health system responsiveness should align to meet the non-clinical needs; such as patient confidentiality, communication and autonomy; by the effective and timely delivery of services in low resource settings (36, 37).

Health service delivery systems that are fragmented, limited and are ineffectively coordinated, with inefficient and separate processes and systems, will diminish resilience (38), effectiveness and efficiency. Health system resilience improves effectiveness and entails activating responsiveness to address health and well-being entwined with socioeconomic determinants; adapting capacity in and beyond health systems to meet community needs and demands; preserving functions and resources in and beyond health systems; and limiting vulnerability to devastating community losses, in health and well-being or finances (34). The effective and efficient elements of health systems allow continuous improvement and higher performance for quality healthcare (Figure 1 and Table 1). Health system effectiveness is aligned with the UHC pillar of quality healthcare. Health system efficiency contributes directly to quality healthcare through continuous improvement and enables more affordable healthcare by resource optimization i.e., operational efficiencies for health service delivery, that from cost-efficiency savings, can reduce healthcare costs thereby allowing greater access—that contributes to the UHC pillars of affordability and accessibility. This helps to prevent financial hardship due to out-of-pocket payments by patients. The robust and resilient elements of health systems provide a supportive and enabling environment for health service delivery (Figure 1 and Table 1). Robust health systems are aligned with quality healthcare by maintaining consistent and improving decent standards of care. Resilient health systems mitigate interruptions to access thereby allowing continuity of care. Responsive and equitable health systems are people-centric and focus and prioritize access to healthcare (Figure 1 and Table 1), in alignment with the UHC pillars of people-centric and accessible healthcare. Responsive health systems are people-centric as they focus on the relevant diseases that afflict people. Equitable health systems are embedded in UHC as they serve to provide access for all. Collectively, these health systems elements gear health systems to deliver people-centric accessible, quality and affordable healthcare towards achieving UHC (Figure 1 and Table 1).

The health systems elements are integral for designing, constructing and configuring health systems for good health service delivery and towards achieving UHC. These elements allow health systems to either be adequately (i.e., sufficient capacity and capability) or optimally (i.e., best possible capacity and capability) geared for service delivery. With the embedding of these elements, health systems are adequately or optimally geared to advance and achieve UHC—affordable, equitable and quality healthcare; detect, prevent and control disease outbreaks; and shield health security thereby promoting economic growth, development and peace (39), in alignment with the SDGs. Further, adequately or optimally geared health systems enable better health service delivery that extends quality care to people. In those LMICs with major resource constraints, designing, constructing, developing, re-configuring and refining health systems, through the adoption and leveraging of these health system elements, are key for advancing UHC. Ultimately, robust and resilient health systems enable greater operational and cost efficiencies and continuity to deliver effective, responsive and equitable quality healthcare—these elements converge to strengthen health systems for the implementation and realization of UHC.

## 6. Conclusions

Countries should use their health resources more efficiently and equitably and can improve efficiency to free up resources for advancing UHC more rapidly (14), interrogate evidence and apply tools for planning strategically, prioritizing equitably and budgeting realistically to realize UHC and other SDG 3 global health targets (13). Efforts should be made to design, construct, re-define and refine health systems that are effective, efficient, equitable, robust, resilient and responsive to adequately or optimally gear health systems to deliver decent healthcare for all.

## Data Availability

The original contributions presented in the study are included in the article/supplementary materials, further inquiries can be directed to the corresponding author.
