# Supplementary material for: Gearing health systems for universal health coverage
Source: Front Health Serv. 2023 Sep 21;3:1200238. doi: 10.3389/frhs.2023.1200238 (PMC10552266; doi:10.3389/frhs.2023.1200238)
Supplement: Supplementary file 1 [file Table1.docx]

**Table S1. Inadequately, adequately and optimally geared health systems**

| **Health system gearing** | **Health resourcing** | **Health service delivery** |
| --- | --- | --- |
| **Inadequate** | Poor | Unable to deliver basic standard of care, i.e. the minimum package of health services, due to poor health resourcing |
| **Adequate** | Sufficient | Provides basic standard of care at the minimal threshold of health resourcing for achieving the minimum package of care |
| **Optimal** | More than sufficient to ample | Exceeds basic standard of care as health resources are optimally utilized |
